# Preventing sepsis in healthcare – 200 years after the birth of Ignaz Semmelweis

**DOI:** 10.2807/1560-7917.ES.2018.23.18.18-00222

**Published:** 2018-05-03

**Authors:** Didier Pittet, Benedetta Allegranzi

**Affiliations:** 1 Infection Control Programme and World Health Organization Collaborating Centre on Patient Safety, University of Geneva Hospitals and Faculty of Medicine, Geneva, Switzerland; 2 Infection Prevention and Control Global Unit, Department of Service Delivery and Safety, World Health Organization (WHO), Geneva, Switzerland

**Keywords:** hand hygiene, Ignaz Semmelweis, sepsis, World Health Organization, infection prevention and control, healthcare-associated infection, maternal mortality, neonatal mortality

The year 2018 marks the 200th anniversary of the birth of Ignaz Philipp Semmelweis, the ‘father’ and pioneer of improved infection prevention and control (IPC) practices. Born on 1 July 1818 in Buda, Hungary, Semmelweis was the son of a respected merchant. His family home was close to Buda Castle where he went to school. Semmelweis initially pursued an arts degree at the university in Pest, a separate town from Buda at the time [1,2], and then moved on to medical studies from 1837, first in Pest, then in Vienna, Austria. In 1846, Semmelweis worked as an assistant in obstetrics at the Vienna General Hospital, one of the largest obstetric clinics in Europe. Apart from working with patients, he was in charge of the supervision of students and medical autopsies.

In the mid-19th century, childbed fever, also called puerperal fever, was a common disease affecting women throughout Europe. The frequently lethal condition was less often acquired by women giving birth at home compared with those delivering in healthcare institutions [3]. The disease was widely attributed to miasma, ‘an emanation or an atmosphere that hovers in the surroundings and causes sickness to those exposed to it by the pervasiveness of its malignant presence’. The high mortality rate among pregnant women, mostly related to infectious diseases and sepsis, was considered to be associated with local cosmo-telluric forces, hygrometric forces, polar currents, or radiation from the constellations [1-3].

At the Vienna General Hospital, pregnant women were admitted to one of two obstetric wards on alternate days. The only difference between the two wards was that one was staffed exclusively by midwives, while in the other ward medical students and doctors were in charge of deliveries and conducted autopsies on dead women in the nearby room. However, mortality from childbed fever was much higher in the latter ward. As soon as he started working in this ward, Semmelweis was struck by the difference [2,4]. The clue to the solution of this enigma was provided by the death of Semmelweis’ friend and colleague, Jakob Kolletschka, who developed a condition resembling childbed fever following a scalpel laceration while supervising an autopsy. This led Semmelweis to hypothesise that the elevated mortality rate in the ward was due to the contamination of the hands of medical students and doctors with ‘cadaverous particles’ during autopsies [1,2,4].

On 15 May 1847, Semmelweis instituted his ground-breaking intervention and ordered everyone to scrub their hands with a chlorinated lime solution when moving from the autopsy room to the delivery room [4]. The impact was immediate and dramatic and soon the mortality rate became comparable between the two wards [1,2,4]. The ‘Semmelweis recipe’ included one part chlorinated lime and 24 parts water, ultimately proven to be extremely effective in annihilating any bacteria [5], much more than soap, and at least comparable with the most effective alcohol-based hand rubs recommended today as the gold standard for patient care [6]. The chlorinated lime solution was chosen by Semmelweis after he had met with the housekeeping staff and looked for the strongest product in use at the hospital: ’it smells, it itches eyes, it’s strong on your hands …it must work!’.

In early 1848, however, a woman with cancer of the uterus with a purulent discharge was examined, and 11 of 12 women examined consecutively in the ward hereafter developed childbirth fever over the next few days [1,2,4]. Semmelweis’ concluded that the disease could be spread from necrotic discharges from living patients as well as autopsy material. From that date onwards, he recommended hand scrubbing also between contacts with each and every patient. These instructions are basis for the present World Health Organization (WHO) concepts behind recommendations on ‘When to hand rub’, and ‘My 5 moments for hand hygiene’ [6,7].

**Figure 1:**
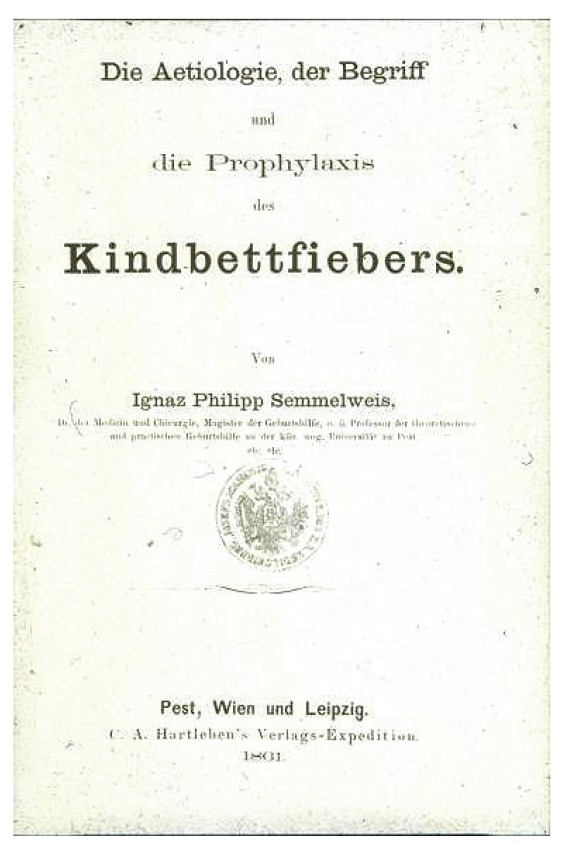
Die Aetiologie, der Begriff und die Prophylaxis des Kindbettfiebers (The aetiology, concept and prophylaxis of childbed fever) by Ignaz Philipp Semmelweis, 1861

Semmelweis’ interventions proved effective in reducing maternal mortality, but they were unpopular among students and colleagues and his contract was not renewed in March 1849 [1,2]. He returned to Budapest, made the same observations, conducted the same intervention with the same impact on patient outcomes, and lost his job once again. In 1861, Semmelweis finally published his seminal work on the aetiology of childbed fever (Figure 1). Sadly, he was committed to a mental asylum in 1865 where he died shortly after admission. The French author and physician, Louis-Ferdinand Céline ended his doctoral dissertation that was dedicated to Semmelweis' work [8] with the sentence: ‘... it would seem that his discovery exceeded the forces of his genius. It was, perhaps, the root cause of all his misfortunes’.

Photo: D. Pittet, reproduced with permission.

Indeed, Semmelweis was a pioneer in scientific risk assessment and in identifying the source of transmission, including conducting an effective intervention. His intervention can be considered as a cluster-randomised controlled trial with random allocation (admission on alternate days) and appropriate long-term follow-up until discharge from the hospital [1-4]. Today, puerperal fever would be classified as maternal sepsis. Sepsis is a life-threatening organ dysfunction caused by a dysregulated host response to infection caused by any type of pathogen that can be acquired either in the community or in healthcare settings [9]. If not recognised early and managed promptly, sepsis can lead to septic shock, multiple organ failure and death. Sepsis remains a public health problem of global concern: more than 30 million people worldwide are estimated to suffer from sepsis each year [10]. One in 10 deaths associated with pregnancy and childbirth is due to sepsis, with more than 95% of deaths due to maternal sepsis occurring in low- and middle-income countries [11]. One million newborn deaths are associated with maternal infection, such as sepsis, each year [12]. Remarkable progress has been made in reducing deaths due to maternal sepsis with global estimates decreasing by 27.7% between 2005 and 2015, but death due to neonatal sepsis is one of only a few remaining exceptions where the reduction has been limited [13]. Although pooled estimates are not available in Europe, mortality reduction from hospital-treated sepsis among children was for example, observed in Germany between 2007 and 2013 (from 124.8 to 104.8 per 100,000 population) [14].

Antimicrobial resistance is a major factor determining clinical unresponsiveness to treatment and a rapid evolution to sepsis and septic shock. Sepsis patients with resistant pathogens have been found to have a higher risk of mortality. For example, less than 50% of children under 5 years of age with acute respiratory infection, diarrhoea or fever receive appropriate care in Africa [15], thus posing them at risk of severe complications, including sepsis. In Europe, carbapenem resistance has been increasing in the last years, in particular among pathogens such as *Klebsiella pneumoniae*, *Pseudomonas aeruginosa* and *Acinetobacter* spp. that often cause healthcare-associated infections [16]; patients infected by carbapenem-resistant pathogens have significantly higher mortality than those affected by carbapenem-susceptible isolates [17,18].

**Figure 2:**
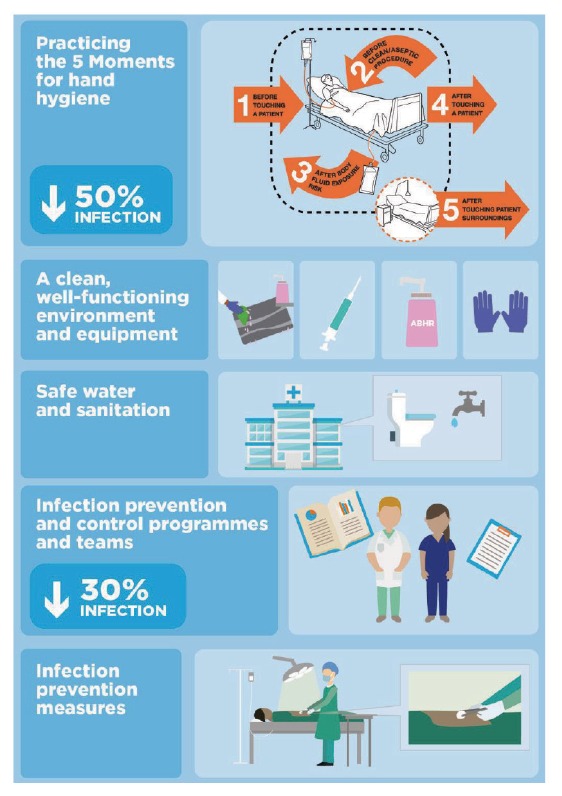
‘How to prevent sepsis in health care’

Sepsis is avoidable in both the community and healthcare settings by preventing infection and its evolution into more severe conditions (Figure 2) [19]. The former is mainly achieved through vaccination, implementation of basic IPC good practices, and improvements in sanitation, nutrition and the delivery of clean water. There is strong evidence to support the positive impact of access to healthcare services, rapid diagnosis, and prompt and appropriate clinical management on infectious disease outcomes. Establishing IPC programmes and teams in hospitals can reduce healthcare-associated infections by at least 30% [20] and, based on Semmelweis’ lesson, hand hygiene alone can reduce them by half [21]. Similarly, handwashing improvements can lead to a 48% reduction of diarrhoeal diseases in the community [22].

Extract from a 2018 World Health Organization Infographic.

**Figure 3:**
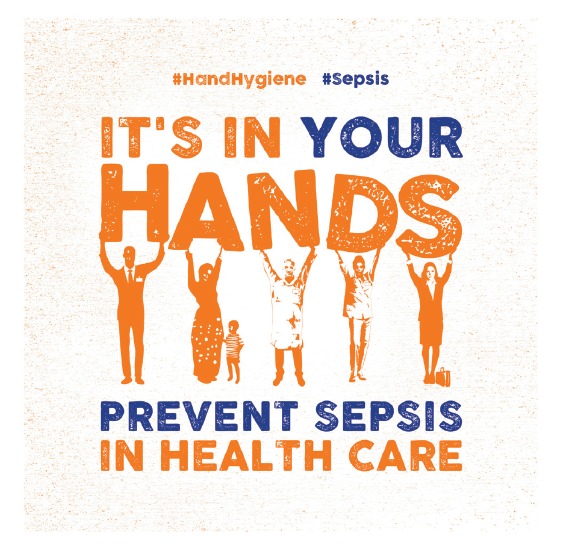
**‘**It’s in your hands; prevent sepsis in health care’

Hand hygiene is the cornerstone of good IPC practices, both in high- and low-resource settings, and it is actively promoted every year on and around 5 May by the WHO ‘SAVE LIVES: Clean Your Hands’ campaign. Over the last decade, more than 20,000 health facilities from over 179 countries have registered for the campaign and many more participate regularly. The focus in 2018 is on the prevention of sepsis with the slogan ‘It’s in your hands – prevent sepsis in health care’ (Figure 3).

5 May 2018 WHO ‘SAVE LIVES: Clean Your Hands’ campaign slogan and main promotional image. 2018 hashtags are: #HandHygiene #Sepsis. Campaign participants are invited to submit photos/selfies holding a board with the slogan and hashtags at www.CleanHandsSaveLives.org.

The calls to action are universal (Table). They invite to join hands and make sure that the commitment to overcoming sepsis is as strong as the exemplary and unmatched commitment of Semmelweis. In this way, each and every one has the possibility to contribute to the implementation of the 2017 World Health Assembly resolution focused on ‘Improving the prevention, diagnosis and clinical management of sepsis’ [23] and to achieving the United Nations Sustainable Development Goals, which target maternal, neonatal and under-5 child mortality.

**Table:** 5 May 2018 WHO ‘SAVE LIVES: Clean Your Hands’ campaign calls to action

Target group	Call to action
**Health workers**	‘Take 5 moments^a^ to clean your hands to prevent sepsis in healthcare’
**IPC leaders**	‘Be a champion in promoting hand hygiene to prevent sepsis in healthcare’
**Health facility leaders**	‘Prevent sepsis in healthcare, make hand hygiene a quality indicator in your hospital’
**Ministries of health**	‘Implement the 2017 WHA sepsis resolution. Make hand hygiene a national marker of healthcare quality’
**Patient advocacy groups**	‘Ask for 5 moments of clean hands to prevent sepsis in healthcare’

IPC: infection prevention and control; WHA: World Health Assembly; WHO: World Health Organization.

^a^ Refers to the ‘My 5 moments for hand hygiene’ as published in the WHO Guidelines on hand hygiene in healthcare [6,7].

To mark the 200th anniversary of Ignaz Semmelweis’ birth, *Eurosurveillance* highlights work on healthcare-associated infections and IPC in a special collection ‘Hospital infection control – 200 years after the birth of Ignaz Semmelweis’.
